# Screening for Celiac Disease in Children with Dental Enamel Defects

**DOI:** 10.5402/2012/763783

**Published:** 2012-06-07

**Authors:** Mostafa Abdel-Aziz El-Hodhod, Iman Ali El-Agouza, Hala Abdel-Al, Noha Samir Kabil, Khaled Abd El-Moez Bayomi

**Affiliations:** ^1^Department of Pediatrics, Faculty of Medicine, Ain Shams University, Cairo 11566, Egypt; ^2^Department of Clinical and Chemical Pathology, Faculty of Medicine, Ain Shams University, Cairo 11566, Egypt; ^3^Department of Orthodontic and Pediatric, Faculty of Dentistry, Ain Shams University, Cairo 11566, Egypt; ^4^Ministry of Health, Cairo, Egypt

## Abstract

*Background*. Dental enamel defects (DEDs) are seen in celiac disease (CD). Aim was to detect frequency of CD among such patients. *Methods*. This study included 140 children with DED. They were tested for CD. Gluten-free diet (GFD) was instituted for CD patients. A cohort of 720, age and sex-matched, normal children represented a control group. Both groups were evaluated clinically. Serum calcium, phosphorus, alkaline phosphatase, serum IgA, and tissue transglutaminase (tTG) IgG and IgA types were measured. *Results*. CD was more diagnosed in patients with DEDs (17.86%) compared to controls (0.97%) (*P* < 0.0001). Majority of nonceliac patients showed grade 1 DED compared to grades 1, 2, and 3 DED in CD. Five children had DED of deciduous teeth and remaining in permanent ones. After 1 year on GFD, DED improved better in CD compared to nonceliac patients. Gastrointestinal symptoms did not vary between celiac and nonceliac DED patients. Lower serum calcium significantly predicted CD in this cohort. *Conclusion*. CD is more prevalent among children with DED than in the general population. These DEDs might be the only manifestation of CD; therefore, screening for CD is highly recommended among those patients especially in presence of underweight and hypocalcemia.

## 1. Introduction

Contrary to early beliefs, celiac disease (CD) is relatively common; however, it still remains underdiagnosed since most cases are atypical, with predominance of extra intestinal manifestations [1].

Available data showed that the prevalence of CD is around 1% of the general population [2]. However, almost 50% of the patients do not present with gastrointestinal symptoms [3]. Thus, in order to identify the greatest number of “atypical” or “silent” CD patients, it has been suggested that the clinicians should investigate those subjects who present with “indirect” signs of CD [4].

As abnormalities of the oral cavity have been reported in CD, clinical examination of the oral cavity can help to identify patients with atypical or silent CD [5, 6]. Specific features of the enamel hypoplasia were suggested as potential clinical markers of diagnosis of celiac disease in suspected cases [7]. Dental enamel defects (DEDs), mainly characterized by pitting, grooving, and sometimes by complete loss of enamel, were first reported in children with CD by Aine et al. [5]. These defects are considered specific to CD if they occur symmetrically and are chronologically distributed in all sections of permanent teeth [7].

Enamel mineralization disturbances secondary to CD do not occur before a period of gluten intake coinciding with enamel mineralization. A possible explanation for the enamel defects could be hypocalcemia or, more likely, a particular genetic condition that leads to a specific immune response to gluten [8]. In addition to hypocalcemia, other systemic factors are associated with enamel hypoplasia, such as malnutrition and vitamin D and A deficiency [9–11]. It is still not clear whether the oral lesions represent a direct manifestation of CD or whether they occur as a result of the indirect effects of malabsorption [12].

The aim of this work was to assess the frequency and predictors of CD among children with dental enamel defects as a step to decide whether these patients are candidates for routine celiac screening.

## 2. Methods

### 2.1. Study Population

This longitudinal clinical study was conducted in the General Pediatric Clinic, Dentistry Pediatric Clinic, and Pediatric Gastroenterology Unit, Children's Hospital, Ain Shams University from May 2008 to April 2011.

The recruitment plan started with diagnosis of DED by the dentists in the general pediatric and pediatric dentistry clinics of Ain Shams University. They were referred to pediatric gastroenterology unit to complete the study provided they fulfilled the inclusion criteria and were free of the exclusion ones.

#### 2.1.1. Inclusion Criteria

1. Patients with DED.
2. Patents age between 4–12 years.

#### 2.1.2. Exclusion Criteria

1. Patients with chronic illness other than gastrointestinal symptoms.
2. Patients on inhalation therapy for bronchial asthma.

The study included 140 patients with DED defined and classified according to Aine et al. [5]. Their age ranged between 4–12 years. They were 72 males and 68 females. They were recruited among attendees of the general and dentistry pediatric clinics who showed any abnormality in teeth structure or shape (1482 children over a 3-year period).

A cohort of 720 healthy, age- (4–12 years), and sex- (371 males and 349 females) matched children was included as a control group. They were recruited among normal children coming for routine checkup in children's hospital in the well child clinic.

### 2.2. Study Procedures

The study was approved by the local ethics committee of the Pediatrics Department, Faculty of Medicine, Ain Shams University. Informed consents were obtained from the legal guardians of the included subjects after explaining the nature of the study to them. This was done once DEDs were diagnosed. Children were subjected to full history taking with special emphasis on dietetic history, gastrointestinal symptoms, dental hygiene, and dentist visits. All included subjects underwent abdominal examination and anthropometric studies (weight and length) and evaluated on WHO growth curves [13].

Oral examination for hard tissue changes (i.e., DED) was done. Examination was carried out at the Pediatric Dentistry Department at Ain Shams Faculty of Dentistry by a pediatric dentist. Most of the patients did not need sedation. Only few patients (below the age of 6 years) required chloral hydrate sedation. In order to avoid masking of defects by dental plaque, teeth were cleaned with a toothbrush [14]. Teeth were carefully evaluated under good artificial light using dental mirrors, dental probes, and sterile gauze without excessive drying. Dental examination was performed in accordance with FDI criteria (modified DDE Index) [15]. The buccal, lingual, and occlusal surfaces were examined. A single defect measuring less than 1 mm in diameter was not recorded. In case of doubt about the existence of a defect, it was scored as normal. Opacities were differentiated from white spot carious lesions based on color, texture, demarcation, and relationship to gingival margin [14]. The enamel defects affecting deciduous and permanent teeth were graded 0 to IV according to Aine's classification [5]. All patients were given full oral hygiene instructions after performing a complete dental prophylaxis. The candidates were followed up for oral hygiene and problematic defects were treated.

Laboratory assessment of celiac disease was based on the quantitative determination of antitissue transglutaminase IgA and IgG (anti-tTG IgA and anti-tTg IgG) using a sandwich Enzyme-Linked Immune-Sorbent Assay (ELISA) kit manufactured by Orgentec, Diagnostika GmbH, (Mainz, Germany). In this technique, anti-tTG IgA and anti-tTG IgG in the samples or standards bind to the microwells coated with human recombinant tTG IgA and tTG IgG. Horseradish peroxidase conjugated to tTG IgA and tTg IgG is added together with its substrate resulting in color development. The intensity of this color, which is proportional to the concentration of anti-tTG IgA and anti-tTg IgG, is measured photometrically at a wavelength of 450 nm. According to the manufacturer's instructions a value above 10 U/mL was used as cutoff value to identify both anti-tTG IgA and anti-tTg IgG positivity.

Total serum IgA was measured, as well, by a radial immunodiffusion method (Diffu-Plate, Biocientífica, Buenos Aires, Argentina). Results were evaluated by using a reference table (routine determination). 5 mg/dL was used as cutoff value to identify IgA deficiency, in which a value below 5 mg/dL was considered to be IgA deficiency [16].

Positive serology (values ranged between 60–120 U/mL) patients (with either one or 2 positive antibodies) were subjected to esophagogastroduodenoscopy and intestinal biopsy from the second part of the duodenum (minimum of 4 biopsies) that were assessed histopathologically for features of celiac disease. The diagnosis of celiac cases was made according to Hill et al. [17]. Celiac patients were put on strict GFD and reassessed after 1 year.

Complete blood count was done on Cell-Dyn-1800 (Abbott Park Illinois, 100 Abbott Park road, 60064-3500 USA). Serum calcium, phosphorus and alkaline phosphatase was done on Synchron Cx9-Pro auto-analyzer (Beckmann instruments Inc. CA, USA).

## 3. Statistical Analysis

The results were collected, tabulated, and statistically analyzed using computer software: SPSS program for Windows, version 12.0.2. It included description of all qualitative variables in the form of frequency and percentage with comparison by *X* ^2^ test. Student *t*-test was used to compare the quantitative variables. Logistic regression model was used to find out the most important independent predictors that could affect certain outcome.

## 4. Results

The study included 140 patients with DED. Their mean age was 8.33 ± 1.92 years. A group of 720 children free from any DEDs were recruited as controls. Their mean age was 8.45 ± 1.73 years. No statistical difference existed between patients and controls as regards age or gender (*P* = 0.691) (*P* = 0.896), respectively. There was a significantly higher percentage of consanguinity among patients with DED (42.86%) compared to controls (23.47%) with *P* < 0.0001.

Of the 140 patients with DEDs, 5 patients showed deciduous teeth abnormality, whereas 135 had the abnormalities in the permanent teeth. None of them showed mixed involvement. Gastrointestinal symptoms did not show statistical difference between patients and controls. However, underweight was significantly more encountered in patients with DEDs than controls (*P* < 0.0001). Celiac disease was more commonly encountered among patients with DEDs compared to controls (*P* < 0.0001) (Table 1).

On comparing children with and without CD among DED patients, the mean weight and height among children with CD (16.6 ± 0.5 kg and 106.5 ± 2.0 cm, resp.) were significantly lower than that of children without CD (29.5 ± 9.7 kg and 123.4 ± 14.0 cm, resp.) with a *P* < 0.001 in both.

There was a significantly lower mean calcium (9.2 ± 0.7 mg/dL) and phosphorous (3.7 ± 0.6 mg/dL) and a higher mean serum alkaline phosphatase (250.2 ± 192.1 IU/L) among cases compared to controls (9.8 ± 0.7 mg/dL, 4.4 ± 0.3 mg/dL and 164.7 ± 26.8 IU/L resp.) with *P* < 0.0001 for all.

No statistically significant difference was found between patients with CD and those without CD as regards gender. Although children with CD had high rates of vomiting, diarrhea, constipation, abdominal pain, and flatulence; these symptoms were not significantly different from those in non-CD patients. The most frequent symptom was abdominal pain (63.6% versus 48.0%), followed by flatulence and diarrhea (45.5% versus 18.0%), vomiting (27.3% versus 13.0%), and constipation (27.3% versus 11.0%) in the celiac and nonceliac children among the case group, respectively.

Consanguinity and underweight were more encountered in patients with CD compared to those without (Table 2). Moreover, underweight was more common in patients with CD more than controls (*X* ^2^= 36.08 and *P* < 0.0001).

There were significantly lower mean serum calcium and higher serum alkaline phosphatase among cases with CD (7.9 ± 0.1 mg/dL and 284.4 ± 199.6 IU/L, resp.) compared to nonceliacs (9.6 ± 0.6 mg/dL and 100.2 ± 15.6 IU/L, resp.), (*P* < 0.0001 and *P* = 0.004, resp.). On the contrary, mean serum phosphorus was not statistically different between CD patients and nonceliacs (3.5 ± 0.8 mg/dL and 3.8 ± 0.6 mg/dL, resp.).

**Figure 1:**
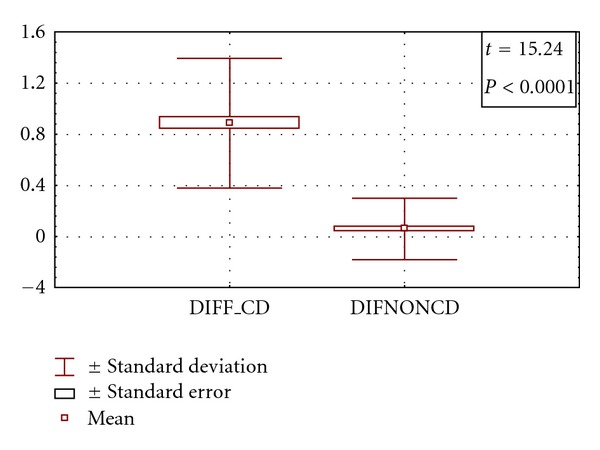
Comparison between the degrees of improvement of grade of DED in patients with CD versus patients with non-CD. (DIFF_CD: degree of improvement in CD, DIFNONCD: degree of improvement in non-CD).

After 1-year followup with routine dental care for all patients in addition to GFD in CD, the frequency of improvement of DED was significantly higher in CD compared to non-CD patients (*P* = 0.0003). Grade 1 pathology was more seen in nonceliac compared to celiac both at start and after 1 year of care (*P* = 0.0001 and *P* = 0.0091, resp.). Grade 2 and 3 pathology were significantly more frequent in CD more than nonceliac patients at the start of study (*P* = 0.0243 and *P* = 0.0452, resp.). However, the difference became insignificant after 1 year of care. Frequency of grade 4 was not different between celiac and nonceliac patients. Moreover, grade 1 was not different in same group before and after 1 year of care (Table 3).  Figure 1 showed that the degree of improvement of grade of DED in celiac patients was significantly higher than that of nonceliac patients. The maximum shift of DED grade was one level. Deciduous teeth showed no improvement at all. 

With regression analysis (at *R* = 0.786 and *R* ^2^ = 0.618), age of patients and hypocalcemia were the only significant determinants (*P* = 0.005 and 0.026, resp.) of the diagnosis of CD among patients with DED, as seen in Table 4. 

## 5. Discussion 

Previous studies focused on description of DED in CD. In fact, this will not reflect how much common is CD among patients presenting with DEDs. In the current study, CD was the underlying cause of DEDs in 17.86% of the studied patients compared to 0.97% of normal children without DEDs. This high frequency justifies consideration of DED patients as candidates for screening for CD. The North American Society for Pediatric Gastroenterology, Hepatology, and Nutrition (NASPGHAN) included the presence of specific dental enamel defects as a risk factor for CD [17]. The same was recommended by Aine et al. and Petrecca et al. [5, 6]. 

Avşar and Kalayci [18] reported that the prevalence of DED in CD subjects was significantly higher (42.2%) than in healthy subjects (9.4%) (*P* < 0.001). Grade 1 type enamel defects were most commonly diagnosed in both groups (20.3% and 6.3%, resp.). 

Wiernik et al. [19] found that 55% celiac patients had DED against 18% control subjects. Similarly, Páez et al. [20] detected DED in 83.3% of the celiac children versus 53.3% of the controls. On the other hand, Procaccini et al. [21] found that the prevalence of enamel hypoplasia was not higher in the study population than in the control group. 

The prevalence of CD in normal children in this cohort was 0.97%, which was a little bit higher than Abu-Zekry et al. [22], who reported a frequency of 0.53 in Egyptian children. The frequency is near to most of western reports (around 1%) [23, 24] and lower than an African report (around 5%) [25]. 

In our study, grades 1, 2, and 3 of DED are more common than grade 4 pathology. Grades 2 and 3 are more common in CD than nonceliac. Improvement of grades, up to normalization of some cases, was significantly more achieved in CD than nonceliac patients who were maintained on routine dental care. The better improvement can be either attributed to direct effect of GFD on enamel or to improved nutritional status after such a regimen. 

Ciacci et al. [26], reported that DEDs were found in 15 patients of transient GFD, 43 of never on GFD and zero of the GFD group. 

The high mean age of CD patients was in agreement of Kuloğlu et al. [27] who reported that the age of children with classical type (7.5 ± 4.3 years) was significantly lower than the age of children with atypical form (10.8 ± 4.3 years). Other studies [28–31] reported a changing pattern in the presentation of pediatric CD towards more predominance of atypical presentations of CD and older age at diagnosis. 

The Celiac Disease Guideline Committee of the NASPGHAN recommended that children and adolescents with symptoms of celiac disease or an increased risk for celiac disease should have a blood test for antibody to tissue transglutaminase, then those with an elevated TTG be referred for an intestinal biopsy to confirm the diagnosis [23]. 

Serum IgA is routinely measured to avoid bias of false-negative tTG IgA type. In our study, there was no difference between children with and without CD as regards serum total IgA level. According to many studies [32–35], selective IgA deficiency should be considered during screening for CD especially with TTG IgA. TTG antibody is recommended as a screening test for CD by many authors [24, 36–38]. 

In our study, consanguinity is evident in 60 children among cases with dental enamel defects (42.86%), while in controls it was 169 (23.47%) with a *P* < 0.0001. Moreover, consanguinity was significantly more encountered in patients with CD (64%) compared to those without celiac pathology (38.26%). It is noticeable that patients with DED without CD still shows significant higher frequency of consanguinity compared to those without DED (*X* ^2^ = 9.35 and *P* = 0.0022). This reflects already high consanguinity rates among normal Egyptian population. It also reflects that a genetic factor may be working in patients with DED with or without CD. 

In patients with CD, serum calcium was significantly lower and serum alkaline phosphatase was significantly higher compared to those without CD. Moreover, serum calcium was the most important predictor of celiac pathology in patients with DED as shown by regression analysis. 

Compared with controls, Praticò et al. [39] stated that celiac patients show at diagnosis a significant increase of serum phosphate and a decrease of calcium level. The authors concluded that CD affects clearly mineral metabolism. Actually, the tendency to hypocalcemia may be attributed to abnormalities of the intestinal mucosa. Similarly, Zanchi et al. [40] reported that calcium and the 25(OH) vitamin D3 levels were lower in children with CD than in control subjects, and the parathyroid hormone level was higher in children with CD than in control subjects. 

In the current study, weight and height were significantly lower in CD patients compared to nonceliac ones. Many studies [27, 41, 42] found that weight and height were below the 3rd percentile in studied CD children. 

In the present study, celiac patients with DEDs had high rates of vomiting, diarrhea, constipation, abdominal pain, and flatulence but difference from nonceliac patients was not significant. Similar to our results, Rashid et al. [38] found that the most frequent symptom was abdominal pain (90.0%) followed by diarrhea (65.0%), vomiting (53.0%), and constipation (30.0%). In contrast to the above-mentioned results, Kuloğlu et al. [27] reported that the most frequent symptom was diarrhea (53.2%) followed by failure to thrive (45.9%), short stature (42.2%), abdominal pain (40.4%), abdominal distention (26.6%), fatigue (27.5%), pallor (23.9%), and vomiting (12.8%). 

 The variable results reported concerning the frequency of symptoms of CD can be explained by the wide spectrum of classical and nonclassical presentations of CD. Also, the difference in population homogeneity, environmental, dietary, and genetic factors may explain the variable results in each study. However, many studies reported that abdominal pain and diarrhea are the most frequent symptoms in classical CD. 

The lack of predictive value of the gastrointestinal manifestations to pick up CD among DED patients is of utmost importance. It is, with the high frequency of CD in this context, a good evidence for a true need for celiac screening among DED patients. 

We can conclude that the prevalence of CD, among children with dental enamel defects, is much higher than in the general population. These enamel problems might be the only manifestation of celiac disease. So, screening for CD is highly recommended among those patients especially in presence of underweight and hypocalcemia.

**What is known?**

Dental enamel defects are common among celiac compared to nonceliac children.

**What is not known?**

To take a look at the other face of the coin, what is the magnitude of CD among patients with dental enamel defects? In other words, is CD common enough in this sector of patients to deserve routine screening?

## Figures and Tables

**Table 1:** Comparison of different variables between patients with dental enamel defects and controls.

	Children with dental	Normal children (720)	*X* ^2^	*P*
	enamel defects (140)			
Males	72 (51.43%)	371 (51.53%)	0.001	*P* = 0.979
Female	68 (48.57%)	349 (48.47%)		

Consanguinity	60 (42.86%)	169 (23.47%)	17.71	*P* < 0.0001
No consanguinity	80 (57.14%)	551 (76.53%)		

Recurrent GI symptoms	25 (17.86%)	146 (20.28%)	0.37	*P* = 0.541
No	115 (82.14%)	574 (79.72)		

Underweight	45 (32.14%)	41 (5.69%)	57.94	*P* < 0.0001
Not underweight	95 (67.86%)	679 (94.31%)		

Celiac	25 (17.86%)	7 (0.97%)	36.95	*P* < 0.0001
Nonceliac	115 (82.14%)	713 (99.03%)		

**Table 2:** Comparison between patients with dental enamel abnormalities who are positive for celiac disease versus those without celiac disease.

	Dental enamel abnormalities	Dental enamel abnormalities	*X* ^2^	*P*
	with celiac disease = 25	without celiac disease = 115		
Males	15 (60%)	60 (52.17%)	0.51	*P* = 0.4770
Female	10 (40%)	55 (47.83%)		

Consanguinity	16 (64%)	44 (38.26%)	5.56	*P* = 0.0184
No consanguinity	9 (36%)	71 (61.74%)		

Recurrent GI symptoms	5 (20%)	20 (17.39%)	0.10	*P* = 0.7576
No	20 (80%)	95 (82.61%)		

Underweight	15 (60%)	30 (26.09%)	10.83	*P* = 0.001
Not underweight	10 (40%)	85 (73.91%)		

**Table 3:** Grading of dental enamel pathology in patients according to celiac positivity and effect of a GFD for 1 year.

	Celiac + DED at	Celiac + DED After	Nonceliac + DED at	Nonceliac + DED	GIa versus GIIa	GIb versus GIIb
	start of study	1 year on GFD	start of study	after 1 year		
	(25)	(25)	(115)	(115)		
	(GIa)	(GIb)	(GIIa)	(GIIb)		
Normal	0	6 (24%)	0	4 (3.48%)		*X* ^2^ = 13.04
						*P* = 0.0003

Grade 1	7 (28%)	7 (28%)	80 (69.57%)	78(67.38%)	*X* ^2^ = 15.08	*X* ^2^ = 6.81
					*P* = 0.0001	*P* = 0.0091
	*X* ^2^ = 0.80 and *P* = 0.3705		*X* ^2^ = .08 and *P* = 0.7761			

Grade 2	10 (40%)	6 (24%)	22 (19.13%)	21 (18.26%)	*X* ^2^ = 5.07	*X* ^2^ = 0.43
					*P* = 0.0243	*P* = 0.5098
	*X* ^2^ = 1.47 and *P* = 0.2253		*X* ^2^ = 0.03 and *P* = 0.8657			

Grade 3	6 (24%)	4(16%)	11 (9.57%)	10 (8.7%)	*X* ^2^ = 4.01	*X* ^2^ = 0.27
					*P* = 0.0452	*P* = 0.6059
	*X* ^2^ = 1.22 and *P* = 0.2695		*X* ^2^ = 0.05 and *P* = 0.8189			

Grade 4	2 (8%)	2 (8%)	2 (1.74%)	2 (1.74%)	*X* ^2^ = 2.90	*X* ^2^ = .44
					*P* = 0.0886	*P* = 0.5066
	*X* ^2^ = 2.08 and *P* = 0.1489		*X* ^2^ = 0.00 and *P* = 1.0000			

**Table 4:** regression summary for possible predictors of celiac disease in patients with DED.

Item	BETA	Standard error of BETA	*B*	Standard error of *B*	*t*	*P*
Age	−0.344	0.117	−0.074	0.025	−2.947	0.005
Consanguinity	−0.191	0.264	−0.090	0.123	−0.725	0.471
Diarrhea	0.158	0.277	0.129	0.227	0.570	0.571
Abdominal distension	−0.040	0.144	−0.032	0.114	−0.277	0.783
Z score for weight	−0.120	0.121	−0.020	0.012	−1.658	0.104
Grade of enamel defects	0.049	0.207	0.043	0.181	0.235	0.815
Dental plaque	−0.209	0.203	−0.170	0.166	−1.026	0.310
Hemoglobin	−0.295	0.305	−0.085	0.088	−0.965	0.339
Calcium level	−0.524	0.228	−0.232	0.101	−2.293	0.026

*R* = 0.78641809, *R*² = 0.61845341, Adjusted *R*² = 0.55112165,  *F*(9,51) = 9.1852,  *P* < 0.000001, Standard Error of estimate = 0.26854.
